# Emergency Management of Medical Wastewater in Hospitals Specializing in Infectious Diseases: A Case Study of Huoshenshan Hospital, Wuhan, China

**DOI:** 10.3390/ijerph19010381

**Published:** 2021-12-30

**Authors:** Jia-Jun He, Shu-Shu Zhao, Hui Zhang, Xia-Ying Liu, Qin Li, Wen-Xuan Fu

**Affiliations:** 1 School of Management, Wuhan Institute of Technology, Wuhan 430205, China; 17070701@wit.edu.cn; 2 School of Chemistry and Environmental Engineering, Wuhan Institute of Technology, Wuhan 430205, China; 22109010117@stu.wit.edu.cn (S.-S.Z.); 22109010085@stu.wit.edu.cn (X.-Y.L.); 22109010089@stu.wit.edu.cn (Q.L.); 22109010124@stu.wit.edu.cn (W.-X.F.); 3 Hubei Engineering Technology Research Center for Chemical Industry Pollution Control, Wuhan Institute of Technology, Wuhan 430205, China

**Keywords:** medical wastewater, emergency management, Huoshenshan Hospital, management measures

## Abstract

Medical wastewater originating from hospitals specializing in infectious diseases pose a major risk to human and environmental health during pandemics. However, there have been few systematic studies on the management of this type of wastewater management. The function of the Huoshenshan Hospital as a designated emergency field hospital for the treatment of COVID-19 has provided lessons for the management measures of medical wastewater, mainly including: (1) Modern information technology, management schemes, and related standard systems provided the legislative foundation for emergency management of medical wastewater. (2) The three-tier prevention and control medical wastewater management system ensured the discharged wastewater met water quality standards, especially for the leak-proof sealed collection system of the first tier, and the biological and chemical treatment technology of the second tier. (3) The establishment of an effective three-tier medical wastewater quality monitoring accountability system. This system was particularly relevant for ensuring continuous data monitoring and dynamic analysis of characteristic indicators. (4) Information disclosure by government and public supervision promoted successful implementation of medical wastewater management and control measures. Public questionnaires (n = 212) further confirmed the effectiveness of information disclosure. The results of this study can act as methodological reference for the emergency management of wastewater in designated infectious disease hospitals under similar situations.

## 1. Introduction

The novel coronavirus disease (COVID-19) first appeared in Wuhan, China, in December 2020, before spreading rapidly worldwide [1]. By the end of December, 2021, there were approximately 280 million cases of COVID-19 cases worldwide, with a mortality rate exceeding 2%. The social, economic, and environmental impacts of COVID-19 have to date far exceeded those of the severe acute respiratory syndrome (SARS) epidemic in 2003 [2]. Hospital wastewater contains hazardous elements and a wide variety of microbial pathogens and viruses [1]. Therefore, this type of wastewater poses a significant risk to human and environment health_._ A recent study showed that the release of partially treated or raw medical wastewater into surface waterways poses a risk of the spread of disease through the “fecal-oral” transmission route [3]. Particularly within the context of the COVID-19 pandemic, appropriate management of medical wastewater is of great significance for minimizing the risks posed to human and environment health [1,4].

President Xi in China responded to the outbreak of COVID-19 in Wuhan by stating: “If Wuhan beats the pandemic, then Hubei province will succeed, and if Hubei defeats it, China will win.” Wuhan succeeded in establishing the Huoshenshan Hospital for the emergency treatment of COVID-19-infected patients, thereby contributing to the curbing the rapid spread of the virus. However, past studies have confirmed that medical wastewater originating from the hospitals contains many pathogenic microorganisms and viruses [1,5]. This type of wastewater is a significant source of pollution and its release into the environment can result in not only degraded water quality but also affect human and environmental health [4]. A recent study showed that that the influent to the wastewater system of the Huoshenshan Hospital was SARS-CoV-2 RNA positive [6]. However, there remains little known on the transmission pathway and mechanism of spread of COVID-19 in medical wastewater. This lack of knowledge increases the difficulty of end-of-pipe treatment of this type of effluent [7]. Studies have shown that collection and pretreatment of medical hospital wastewater greatly reduces environmental risk [8]. Therefore, it is essential that medical wastewater containing SARS coronavirus 2 (CoV-2) be regulated and treated at the source to allow the effective control of the transmission for the improvement of the medical wastewater management system. Some examples of subsequent medical wastewater management systems that have been implemented include the Standards for the Discharge of Water Pollutants from Medical Organizations (GB 18466-2005) [9], Engineering and Technical Specifications for Hospital Wastewater Treatment Engineering (HJ 2029-2013) [10], and General Rules for Disinfection of Sites of Epidemic Origin (GB 19193-2015) [11]. These management systems stipulate the principles, processes, methods, and measures for the treatment and the limits of discharged pollutants for wastewater originating from hospitals treating infectious diseases. On the basis of lessons learned from the SARS outbreak regarding the building and environmental pollution problems caused by inadequate disposal of medical waste [12], Huoshenshan Hospital was successfully built up using Chinese speed with a reference on architectural patterns of Xiaotangshan hospital [13], while simultaneously constructing wastewater treatment facilities and online monitoring stations to support wastewater treatment capacity [14]. Various new legislation measures for emergency monitoring and treatment have been proposed and implemented to effectively utilize the medical wastewater treatment monitoring facilities and equipment while minimizing health and environmental risks associated with wastewater [15,16,17,18]. Some examples include the Emergency Monitoring Program in response to the COVID-19 Outbreak and the Technical Program for Emergency Treatment of Medical Wastewater Contaminated with Novel Coronavirus [17]. Many countries have traditionally treated medical wastewater, which was collected, together with urban wastewater to a wastewater treatment plant, in many countries [1]. The present study proposed a three-tier (collection, disposal and discharge) prevention and control management system to effectively eliminate the risk posed by medical wastewater on environment and human health. Three-tier management of hospital wastewater can ensure that discharged treated wastewater meets corresponding water quality standards. This can be attributed to the implementation of multiple strict processes, such as the double intensified disinfection and moving bed biofilm reactor (MBBR) biological and chemical treatment at the second level [19]. This three-tier management model can be applied for the disposal of medical wastewater globally. Monitoring data showed that the surface water quality in Wuhan was not negatively affected during the outbreak [20]. However, there remains a lack of effective emergency regulation and treatment for medical wastewater from hospitals treating infectious diseases. The successful regulation and treatment medical wastewater originating from the Huoshenshan Hospital can provide a paradigm for treating medical wastewater bearing pathogens.

Although there have been a variety of recent studies on the management of hospital wastewater, there remains no systematic study on the emergency management of medical wastewater from hospitals treating infectious disease. The aim of the present study was to explore emergency medical wastewater management measures based on the Huoshenshan Hospital as a case study. The present study collated the experience gained within the regulation and management of medical wastewater under the following four categories: (1) medical wastewater treatment technology; (2) environmental monitoring measures; (3) the legislative foundation for wastewater emergency treatment; and (4) information disclosure and public opinions. The results of the present study can provide a reference for the emergency regulation and management of hospital wastewater bearing pathogens.

## 2. Methods and Data Resource

The Chinese government decided to rapidly construct two temporary field hospitals during the COVID-19 pandemic to ease the burden on the local hospitals. The Wuhan Huoshenshan Hospital was constructed in just nine days, three days less than the time required to construct the Leishenshan Hospital [21]. The Huoshenshan Hospital was also the first field hospital globally to be constructed to care for COVID-19 patients. Considering the global spread of COVID-19 and the serious shortage of medical supplies, and the importance of medical wastewater management in many countries and region, the lessons and experience of Huoshenshan Hospital in China are expected to provide valuable information for other countries and regions to assist in combating the COVID-19 outbreak. Therefore, the present study chose Huoshenshan Hospital as a case study.

The present study combined qualitative and quantitative information on the management of medical wastewater by Huoshenshan Hospital emergency management measures of medical wastewater were systemically summarized. Information was obtained from three categories of data resources; (1) major media sources; (2) official information; and (3) published studies. Information relating to the construction of the Huoshenshan Hospital and the three-tier environmental management of medical wastewater were collected mainly from major media sources (e.g., Xinhua News, Beijing Youth Daily, China Environmental News, Global Times, etc.). Information was obtained from a total of 14 media sources, accounting for 29% of the total references. Information on the relevant regulations and policies for the emergency treatment of medical wastewater and environmental monitoring data were obtained from official report and statistical data (11 references), including the Wuhan Bureau of Ecological Environment, and websites of related authorities (e.g., the CCTV international online and the Ministry of Ecology and Environment). Recent published studies were analyzed to obtain information on the health and environmental risks of medical wastewater, other studies’ management system, and further highlighted the necessity and effectiveness of emergency measure of medical wastewater for the case study.

As an important management lesson, such as public participation regarding the case study, its basic information was obtained via questionnaire survey. The questionnaire was structured to understand the extent of information disclosure and to facilitate public participation, There were eight questions in the questionnaire, which could be categorized into three parts: (1) a basic characterization of the respondent (i.e., ages, regions, education level and occupation, Q1 to Q4; (2) the behavior of the respondents in relation to public participation, Q5; (3) the means of public participation employed by the respondent and its context within the case study, Q6 to Q8. Online questionnaires were randomly distributed in May 2020 via a WeChat group and a Q chat group among friends, students, and colleagues. In total, data information was analyzed from 212 individuals of different ages, regions, educational levels, and occupations using Microsoft Excel (Microsoft Corporation. Redmond, WA, USA).

## 3. Results and Discussion 

The present study systemically summarized the process of construction of the Huoshenshan Hospital and its successful environmental management of medical wastewater based on the three categories of information (major media sources, official information, and published studies). Section 3.1, Section 3.2, Section 3.3, Section 3.4 and Section 3.5 outline the lessons learnt.

### 3.1. Construction Progress of the Huoshenshan Hospital

**Figure 1:**
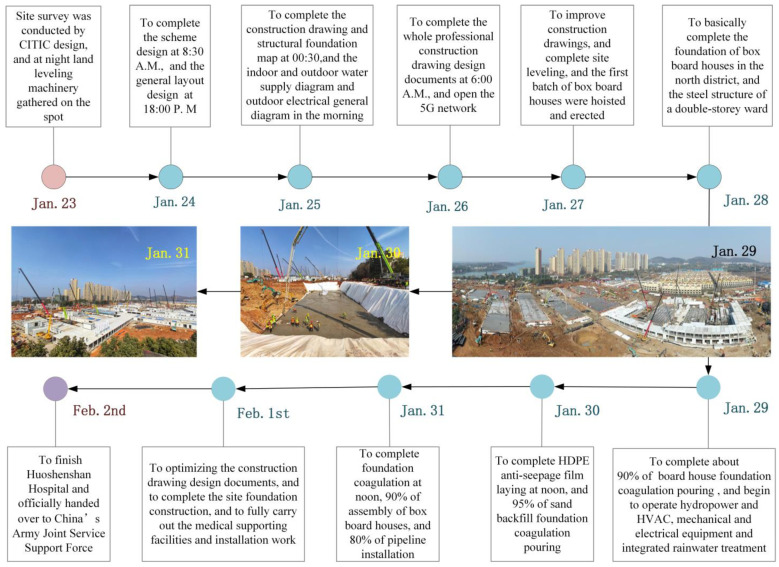
Timeline of progress in the construction progress of the Huoshenshan Hospital, Wuhan, China, between January and February, 2020. Images were taken from the official website of the CITIC General Institute of Architectural Design & Research Co., with permission.

The design of the hospital was initiated just three days after Wuhan went into lockdown. Construction of the hospital, as well as the laying down of high-density polyethylene (HDPE) anti-seepage film for wastewater, was completed by noon of the 29 January 2020. All medical supporting facilities were installed by the 1 February 2020, and the hospital was officially handed over to China’s Army Joint Service on the following day [18,21]. Figure 1 shows a timeline of progress of the construction of the Huoshenshan Hospital. The hospital was situated in a sanatorium area near Zhiyin Lake in southwestern Wuhan, covered an area of ~34,000 square meters, and had a capacity of 1000 beds. The hospital utilized modern information technology including a 5th Generation (5G) Mobile Network, Artificial Intelligence (AI), and Internet of Things (IoT) to achieve intelligent security, remote medical consultation, intelligent reviews of medical records, and “contactless” operation and maintenance [22]. For example, 5G coverage and wired broadband were utilized to connect to a remote consultation platform that allowed experienced doctors in other provinces around China to conduct remote consultations with patients in Wuhan [23].

### 3.2. Development of Relevant Regulations and Policies 

As shown in Table 1, the design of wastewater treatment facilities at the Huoshenshan Hospital during the pandemic was in strict accordance with existing specifications (HJ 2029–2013) and standards (GB 18466–2005). In particular, the design of the wastewater treatment facilities was in adherence to the Standards for the Discharge of Water Pollutants from Medical Organizations. These standards regulate medical wastewater discharge to limit the release of toxins and pathogens into the environment, such as fecal coliform bacteria, total residual chlorine, etc. [24]. The legislative branch in China responded quickly to the COVID-19 pandemic by improving the legal structure of the public emergency services. In particular, the regulations relating to wastewater discharged from hospitals were updated to consider the risk posed by infectious diseases. Prior to the COVID-19 pandemic, medical wastewater in China was discharged directly into the municipal sewer system, with medical wastewater allowed to mix with municipal wastewater, and this mixture of wastewater treated at a municipal wastewater treatment plant, following a regulation and management system similar to those practiced in some developed countries, such as Australia and Spain [25,26]. However, the COVID-19 pandemic motivated the Ministry of Ecology and Environment to apply the Internet/AI technology (mentioned in Section 3.1) for the construction of an online communication platform. This platform allowed remote communication among relevant experts, front-line operators, and ecological and environmental management personnel [27]. This improved remote communication and allowed experts groups to provide online technical guidance for the management of medical wastewater of the online communication platform based on Internet/AI technology, including its disinfection and disposal and equipment operation [28]. Meanwhile, the online platform facilitated the development and prompt release of the Notice, the Technical Program, and the Monitoring Program upon the completion of the Huoshenshan Hospital based on the experience of medical wastewater regulation and management obtained since the SARS outbreak. These legal documents proposed the legislative requirements for the regulation and management of medical wastewater at the source. These management aspects included the classification, collection, storage, and treatment of wastewater, as well as guidance for emergency monitoring of variables, such as residual chlorine and biological toxicity. The documents also considered measures for the protection from and control of pathogen-bearing wastewater. The requirements specified the characteristics of COVID-19 indicators for use in monitoring and disinfection, as well as sterilization treatment criteria, to achieve the effective management of the medical wastewater produced by the Huoshenshan Hospital within the shortest time frame.

### 3.3. Three-Tier Prevention and Control System 

**Figure 2:**
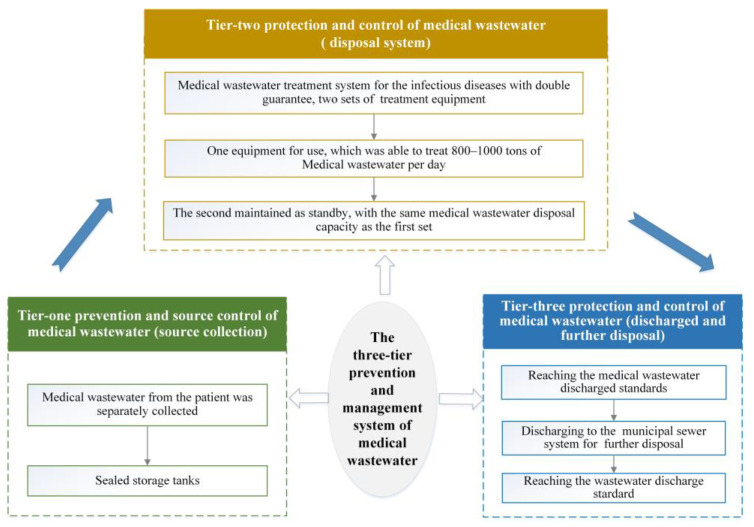
A conceptual diagram illustrating the three-tier system for the management of medical wastewater.

Figure 2 shows the three-tier system for the management of medical wastewater. The system facilitates the systematic treatment of wastewater, as well as the management of routes of disease transmission during outbreaks of infectious diseases. The system stipulates strict separation of medical wastewater and collection at the source, specialized treatment at different levels, and discharge to specified standards. 

#### 3.3.1. Tier-One Prevention and Source Control (Source Collection)

Medical wastewater carrying SARS-CoV-2 increases the risks of continuous disease transmission and spread of the viruses [30]. Therefore, higher standards need to be adopted for the treatment of wastewater generated by hospitals treating infectious diseases than those for ordinary hospitals. The tier-one prevention and control system should act to prevent and control the source. Wastewater generated at the Huoshenshan Hospital was physically isolated and collected in a fully enclosed manner to prevent exposure of wastewater to the ambient air to the soil [31]. A layer of high-density polyethylene (HDPE) film was installed into the foundation of the hospital to prevent seeping and to ensure physical isolation of the aboveground structures from groundwater and soil. Domestic sewage and medical wastewater from the infectious disease ward were collected separately, and medical wastewater was delivered separately, in sealed storage tanks.

#### 3.3.2. Tier-Two Protection and Control (Disposal System)

**Figure 3:**
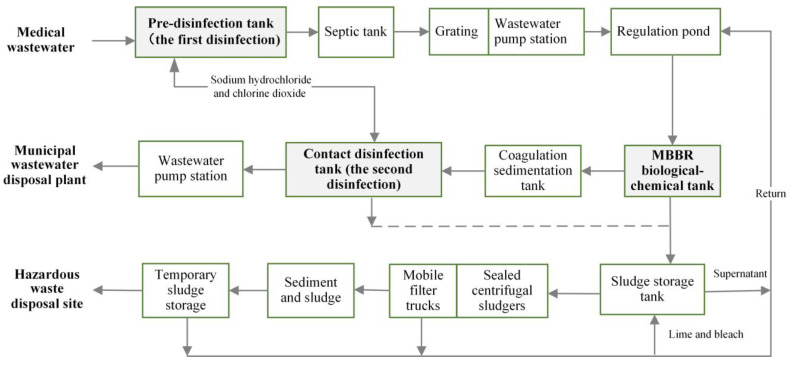
Treatment process of medical wastewater for Huoshenshan Hospital.

The wastewater in the sealed storage tanks at the hospital was then transported from the hospital through a special pipeline into the medical wastewater treatment system. This treatment system was a tier-two prevention and control wastewater disposal system. The successful design and construction of this system were viewed as a case study in emergency management of medical wastewater. Two sets of equipment required for the treatment of wastewater during this period were installed, with one set maintained as standby to increase the resilience of the system. A single device was able to treat 800–1000 tons of wastewater per day [16]. As shown in Figure 3, the tier-two protection and control system incorporated multiple processes, including pre-disinfection, a septic tank, a conditioning tank, biological and chemical treatment (degradation of chemical oxygen demand (COD) and ammonia nitrogen (ANH_3_-N)), sedimentation, and (secondary) disinfection. The experience gained in medical wastewater treatment during the SARS pandemic, in combination with reference “HJ 2029-2013”, allowed the effective treatment of fecal coliforms and SARS–CoV by disinfection, and the Hospital implemented a safe, effective, and cost-effective two-level intensified disinfection system [4]. A pre-disinfection-septic tank-disinfection tank (secondary disinfection) [24], lasting for 5 h, far exceeds the national standard of 1.5 h [14]. This standard has been incorporated into the nationally implemented Design Guidelines for Emergency Treatment Facilities for COVID-19 (Trial) and the Design Standards for Emergency Medical Facilities for COVID-19 (T/CECS 661-2020) issued by the China Association for Engineering Construction Standardization.

Anaerobic degradation in the septic tank generally went through acidogenesis and methanogenesis, during which organic wastewater and sewage with high concentrations of COD and ANH_3_-N were treated. This process produced a low quantity of surplus sludge, which was easily concentrated and dewatered, during which the sludge was also disinfected. The automatic mechanical screen removed debris to avoid being negatively affected, such as tissue paper products [14]. The activated sludge method is a commonly applied biological treatment process, which allows the removal of organic pollutants in colloidal and dissolved forms in wastewater. Degraded organic matter and non-degraded pollutants existed in the sludge and were separated from the water through solid-liquid separation. The wastewater was disinfected to reach the specified pretreatment standard. Table 2 shows the design effluent quality of medical wastewater. The moving bed biofilm reactor (MBBR) process utilizes the traditional fluidized bed and contact oxidation process to effectively eliminate organic pollutants [21]. The operation of the process was also simple, with resistance to shock loads and low costs [32]. Therefore, MBBR was selected as the biological and chemical treatment technology used in the hospital [21]. The sludge generated from the wastewater treatment was concentrated and dewatered, following which it was transported to a centralized location for disposal. The odor (e.g., ammonia, chlorine, hydrogen sulfide gas) from the wastewater treatment station was collected, disinfected, and deodorize to finally meet standard GB18466-2005, following which it was discharged [33]. The sludge storage tank was disinfected with lime and bleach, and the sludge was removed using sealed centrifugal sludgers and mobile filter trucks. After meeting standard (GB18466-2005), the sludge was sealed and transported to a hazardous waste disposal site and disposed through incineration as hazardous waste [31].

The wastewater treatment facilities at the Huoshenshan Hospital were equipped with online monitoring stations for dynamic tracking of the management and control of wastewater quality. Eco-environment division personnel conducted daily on-site inspection of pollution control facilities, during which they assessed the discharge and monitoring data [21,31]. When discharge did not meet the specified standards, a sequential investigation was conducted to identify and solve the problems. The strict monitoring and control measures implemented achieved a certain degree of success. The results of monitoring of hospital wastewater quality showed that the levels of the characteristic indicators, such as residual chlorine and fecal coliform, were generally in conformance with the limits set in the Standards for the Discharge of Water Pollutants from Medical Organizations (GB 18466-2005) (Table 2) from 1–21 February 2020. During the hospital operation stage from 2 February to the close of the hospital, the levels of characteristic indicators of the effluent at the outlet of a contact disinfection tank were maintained within the standard range [34], e.g., a specified standard (GB 18466-2005) for the total residual chlorine (Table 2).

#### 3.3.3. Tier-Three Protection and Control (Discharge and Further Disposal of Wastewater)

The treated wastewater was tested by the online monitoring station to ensure that the water quality of wastewater met the standards (GB18466-2005) and technical specifications (HJ 2019-2013) shown in Table 2. This treated medical wastewater was then discharged into the municipal sewer system, finally reaching the municipal wastewater treatment plant (Shiyang Wastewater Treatment Plant). This is the tier-three prevention and control measure. The municipal wastewater treatment plants further treated the wastewater by means of the activated sludge method to ensure that the produced treated effluent reached the standard for pollutants discharged by urban wastewater treatment plants (GB 18918-2002) [35,36].

### 3.4. Measures for the Management of Environmental Monitoring 

The Ministry of Ecology and Environment focused on the environmental monitoring of designated hospitals in Wuhan during the pandemic and swiftly formulated a Monitoring Program on 30 January 2021 (Table 1). This program specified the emergency monitoring of characteristic pollutants for pandemic prevention and control, including for residual chlorine and biological toxicity.

**Figure 4:**
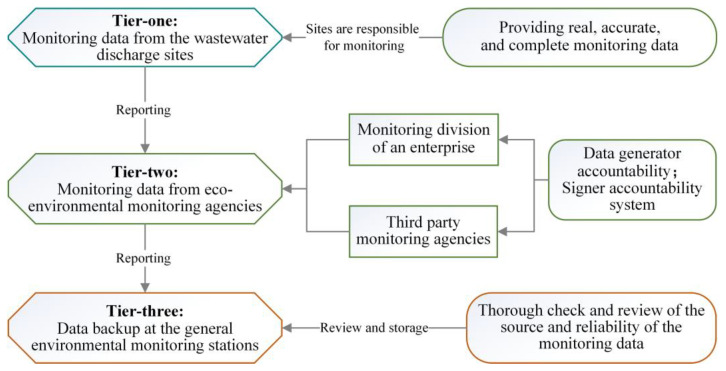
A three-tier accountability system for monitoring hospital wastewater treatment units.

The Wuhan Environmental Monitoring Center used a field investigation to rapidly draft the Emergency Monitoring Program for COVID-19 Outbreak in Wuhan (hereinafter referred to as the “Wuhan Monitoring Program”) [38]. The center also mobilized relevant enterprises and monitoring units to provide real-time dynamic monitoring of the medical wastewater produced by 63 designated hospitals in the city, including the Huoshenshan Hospital, according to the program [27,37]. Monitoring of the medical wastewater of the designated hospitals (including Huoshenshan Hospital) conformed to the requirements of the Wuhan Monitoring Program. The monitoring program followed the three-tier accountability system of data monitoring, recording, and storage. Additionally, the Environmental Emergency Monitoring Information Briefing conducted the pandemic in Wuhan was prepared and submitted to the higher-level monitoring authority for verification and record keeping. Environmental monitoring of medical wastewater quality during the epidemic also promoted the development of ecological and environmental big data platforms. A three-tier accountability system for monitoring the data collected from wastewater units was developed. General environmental monitoring stations (Figure 4) were established, and the legislative, scientific, and ethical foundations of the environmental monitoring system were constructed.

### 3.5. Importance of Environmental Governance 

#### 3.5.1. Information Disclosure and Public Participation

With the development of internet technology, Chinese netizens have been playing an increasingly substantial role in public opinion supervision and management of environmental protection [39]. The 46th Statistical Report on the Development of Internet in China by the China Internet Network Information Center (CNNIC)found that as of June 2020. Internet users in China has reached 940 million. This represents an Internet penetration rate of 67.0%. Mobile users accounted for 99.2% of the total Internet users, thereby providing a solid foundation for receiving and publishing information. The Ministry of Environmental Protection promulgated the Measures for Public Participation in Environmental Protection (Table 3) on 13 June 2015. These measures encouraged and facilitated citizens, legal persons, and organizations in making their public opinion known and also facilitated social supervisions of public environmental affairs by legal means.

Information disclosure by government agencies is an important means of providing information to the general public. The dissemination of emergency information (information disclosure) online has become an active means of expressing public opinion, further boosting public participation [40]. During the COVID-19 pandemic, the Ministry of Ecology and Environment disseminated environmental information relating to the emergency to the public in accordance with the laws [5]. This information included details of the medical wastewater generated by the Huoshenshan Hospital and provided accurate and timely information to the public and social organizations by various forms of media and through the official website [38,41,42]. This disclosure of information ensured that information on the process of the construction of the medical wastewater treatment facilities and medical wastewater treatment data of the Huoshenshan Hospital were open and transparent.

#### 3.5.2. Survey on Public Participation within the Development and Operation of the Huoshenshan Hospital

The release of information on the COVID-19 pandemic has been timely, accurate, open, and transparent. Netizens received not only the open and transparent release of pandemic information but were also active supporters and protectors of this policy [41]. Information on the construction of the Huoshenshan Hospital was released online by government and state media [41,42], and public participation became an important driver for the orderly progression of the construction of the hospital and its associated wastewater treatment management measures. The present study conducted a questionnaire survey to understand the breadth and depth of information disclosure on the hospital and its medical wastewater management that was disclosed, as well as the extent of public participation.

**Figure 5:**
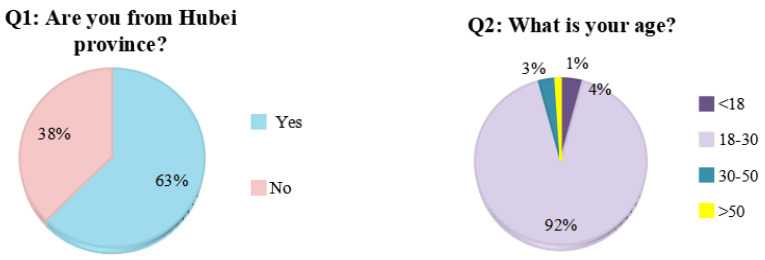

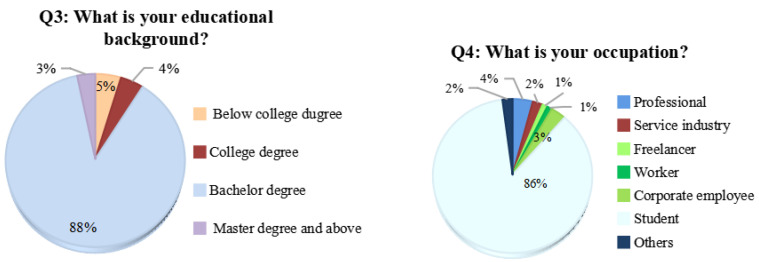
Characterization of survey respondents.

As shown in Figure 5, most respondents were from Hubei Province, the nearest province to the case study. The respondents were representative of a wide range of ages, educational backgrounds, and occupations, although the majority of respondents were students between 18–30 years with a bachelor degree. 

**Figure 6:**
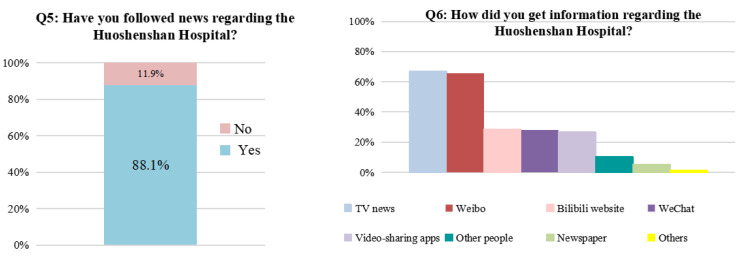
Survey on public interest in information released for the construction and operation of Huoshenshan Hospital.

The findings showed that most of the population (88%) had paid close attention to the development of the Huoshenshan Hospital (Figure 6). The respondents indicated that they had obtained information on the hospital through multiple channels, including TV news, social media, such as Weibo and WeChat, and Apps providing short videos to mobile users. This informed the audience of information disclosure. Most of the public was able to actively utilize the well-developed Internet and a variety of web tools to receive the widely disseminated information on the management of medical wastewater produced by the case hospital (Figure 6). This openness of information ensured enthusiasm and a degree of participation by the public within the construction of the hospital and the management of its wastewater to an extent. Concurrently, the requirement for broad public participation in the Measures for Environmental Information Disclosure (Trial) has been fulfilled.

**Figure 7:**
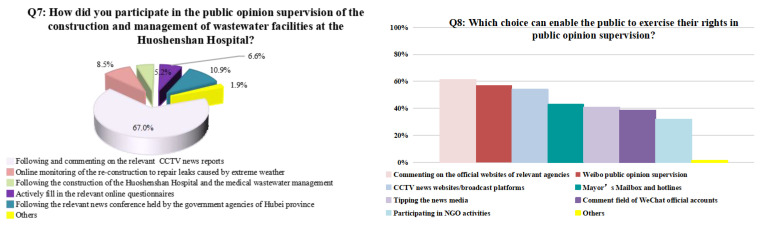
Survey on how the general public exercised their rights in public opinion supervision.

The survey asked the respondents to specify the primary way they used to remain informed and to express their opinion on the construction of wastewater facilities and the management of wastewater at the Huoshenshan Hospital. Most respondents (67%) indicated that they participated through following the news and comments on CCTV reports (Figure 7). The live broadcast of CCTV attracted 50 million “cloud supervisors” during the peak viewing times [43]. Overseas netizens were also able to simultaneously watched the broadcast through YouTube and other foreign video websites [44]. The survey results revealed the enthusiasm, positivity, and high levels of awareness of the public regarding construction and operation of the hospital. The survey results also showed that connectivity of the current society allowed the general public to receive transparent information through a variety of avenues. This allowed public opinion to supervise the construction and operation of the hospital through a variety of real-time, dynamic, and prompt approaches, including publishing comments on the official websites of relevant agencies, Weibo, and CCTV news websites/live broadcast platforms, contacting government officials through mails and hotlines, and commenting at WeChat official accounts.

#### 3.5.3. Comparison with Developed Countries

Disclosure of environmental information Disclosure represents a new tool for environmental governance in the era of big data and information [45]. Developed countries, such as the European Union (EU) member states, the United States (U.S.), and Japan, have actively promoted public participation and have established related legal systems to guarantee and emphasize the importance of public participation and social supervision [46]. For example, the U.S. passed the National Environmental Policy Act as early as 1969, which represented the first established the status and rights of the public to participate in environmental impact assessment. The Basic Environment Law of Japan enacted in 1993 specified the avenues, procedures, and means of public participation. The Environmental Impact Assessment Law of 2003 of China defined public participation in environmental impact assessment of construction projects. Some recent studies have shown the positive effect of public participation in and supervision of environmental management for the mitigation of environmental degradation [47]. Although public legislation for public participation of China has been gradually improving, there remains a demand for legislative and executive guarantees for public participation and environmental supervision for specific industries. To some extent, these gaps in legislation have impacted the extent of public participation. For example, there remains no legislation related to public participation in the management of medical wastewater. Therefore, the case study on the construction of the Huoshenshan Hospital and the management of its wastewater can act as a reference for facilitating the participation of netizens in supervision of environmental management though public opinion through a variety of network platforms and information tools. The results of the present study showed a rapid progression in the process compared to that during the SARS epidemic period. Achieving the positive effect of public opinion supervision of environmental management through public opinion has two requirements. First, the disclosure and transparency of information needs to be improved. Secondly, public awareness should be enhanced and enthusiasm and motivation of netizens and non-governmental organizations (NGOs) should be promoted to ultimately mobilize the public to take part in environmental management.

## 4. Limitation and Further Study

The present study had certain limitations, particularly regarding the survey on the public participation based on the questionnaire method. First, only 14% of respondents were not students. This possibly has impacted the representativeness of the designed respondents to some extent. Second, the questions in the survey mainly focused on the information disclosure and the means of public participation, thereby possibly limiting the accuracy of information on public participation. More accurate information on public participation can be obtained in a future study by increasing the range of occupations among respondents, adding problems designed in the questionnaire, such as public opinion on the quality of information received (timeliness, detail, ease of access, understandability, comprehension, etc.) and the levels of public confidence regarding any actions taken in response to their comments/suggestions.

## 5. Conclusions

The present study aimed to provide a roadmap for the emergency management of medical wastewater in the future. Effective management measures for the medical wastewater originating from hospitals treating infectious diseases, using the Huoshenshan Hospital as a case study, were systematically summarized based on three types of data resources, namely media sources, official information, and published articles. The main findings were as follows:

(1) The successful and rapid construction of a modern emergency hospital with modern information technology, including 5G, AI, and IoT, occurred concurrently with the construction of effective wastewater management equipment, including an underground HDPE film layer for the isolation of source’s wastewater.
(2) The three-tier protection and control system was implemented for the emergency treatment of wastewater. The system was implemented by classification and collection of the medical wastewater from the ward, implementation of hospital wastewater treatment facilities, and municipal sewer system. Tier one focused on the prevention of pollution prevention at the source; tier two concentrated on the disinfection of the wastewater and the biological and chemical treatment of organic pollutants, to ensure that discharged wastewater met the water quality standards.
(3) The management of monitoring aimed to ensure effective operation of monitoring equipment, standardization of monitoring process, and the provision of timely, reliable, verifiable, and recordable monitoring data. The three-tier accountability system within the data monitoring process guaranteed accuracy of monitoring data and also promoted the construction and development of big data platforms.
(4) Medical Wastewater prevention and control technologies and measures were effectively implemented through the emergency legislative guarantee for the collection, monitoring, treatment, and discharge of the medical wastewater, as well as the modern information techniques.
(5) The results of the questionnaire survey confirmed that the most of the population follow updates on the Huoshenshan Hospital through online tools, such as TV news, Weibo, WeChat, and video-sharing apps for mobile users. The public participated in online reviews and surveys of medical wastewater treatment facilities and management issues. Disclosure of environmental information should be strengthened, and public participation in industry-specific legislation should be improved, to promote the supervision of the management and control of medical wastewater through public opinion.

## Figures and Tables

**Table 1:** A summary of regulations relating to management of medical wastewater in China.

Laws, Regulations, and Standards	Issuing Authority	Time of Issuance	Relevant Provisions of Wastewater Management/Reference
Technical Guideline for Hospital Wastewater Treatment (Environment Development (2003) No. 197)	Ministry of Ecology and Environment	1 December 2013	Separate medical wastewater from non-medical wastewater by using separate different piping systems; required mandatory septic tanks and secondary treatment in hospitals treating infectious diseases; required pre-disinfection [29]
Standards for the Discharge of Water Pollutants from Medical Organizations (The Standards, GB 18466-2005)	Ministry of Ecology and Environment	17 May 2005	Specify the pollutant discharge limits for wastewater produced by hospitals treating infectious diseases [9]
Engineering and Technical Specifications for Hospital Wastewater Treatment Engineering (The Specifications, HJ 2029-2013)	Ministry of Ecology and Environment	29 March 2013	Specify the wastewater treatment process and disinfection measures for medical wastewater produced by hospitals treating infectious diseases [10]
General Rules for Disinfection of Sites of Epidemic Origin (GB 19193-2015)	General Administration of Quality Supervision, Inspection and Quarantine	12 June 2015	Specify the requirements, principles, and effects of disinfection of sites of epidemic origin [11]
Emergency Monitoring Program in response to COVID-19 Outbreak (The Monitoring Program)	Ministry of Ecology and Environment	31 January 2020	Specify the measures for emergency monitoring of characteristic pollutants for pandemic prevention and control, such as residual chlorine and biological toxicity [15]
Notice on the Regulations of Medical Wastewater and Municipal Wastewater from the COVID-19 Outbreak (The Notice)	Ministry of Ecology and Environment	31 January 2020	Specify the classification and collection, monitoring, treatment, and discharge requirements for medical wastewater at the site of the outbreak [16]
Technical Program for Emergency Treatment of Medical Wastewater Contaminated with Novel Coronavirus (Trial)” (The Technical Program)	Ministry of Ecology and Environment	1 February 2020	Strengthen the classification and management of wastewater and enhance disinfection and sterilization to meet the discharge standards and ultimately prevent the spread of viruses [17]
Ecological Environmental Monitoring Regulations (Draft) (The Monitoring Draft)	Ministry of Ecology and Environment	17 February 2020	Identify the responsible monitoring parties, and monitoring data and strengthen monitoring, reporting, and reviewing [18]

**Table 2:** Medical wastewater associated with standard, influent, and effluent quality.

No	Item	Standard Value	Design Influent & Effluent Quality of Medical Wastewater		Actual Effluent Quality of Medical Wastewater
			Influent Quality	Effluent Quality	
1	Number of fecal coliforms	≤100 MPN.L^−1^	≤3.0 × 10^8^ MPN.L^−1^	≤5000 MPN.L^−1^	≤100
2	Intestinal bacteria	ND	---	---	---
3	Tubercule bacilli	ND	---		---
4	pH value	6–9	6–9	6–9	7.9 ± 0.7
5	COD	≤60 mg L^−1^	≤350 mg L^−1^	≤250 mg L^−1^	40.66 ± 5.23
6	BOD	≤20 mg L^−1^	≤150 mg L^−1^	≤100 mg L^−1^	---
7	SS	≤20 mg L^−1^	≤120 mg L^−1^	≤60 mg L^−1^	---
8	ANH_3_-N	≤15 mg L^−1^	≤30 mg L^−1^	---	1.53 ± 0.38
9	The total residual chlorine	6.5~10 mg L^−1^	---	---	6.5~10 mg L^−1^
10	Animal and vegetable oil	≤5 mg L^−1^	≤50 mg L^−1^	≤20 mg L^−1^	---
11	SARS-CoV-2	ND	---	ND	---

Note: data, reference [31,37]; BOD–biological oxygen demand; COD–chemical oxygen demand; SS–suspended solids; sign ---, vailable; ND, cannot be detected.

**Table 3:** Laws, measures, and regulations regarding the disclosure of environmental safety information to the public in China.

Name	Issuing Authority	Issuing Date	Remarks
Emergency Regulations for Public Health Emergencies	State Council	29 May 2003	To establish a system of prompt notification and timely information release
Measures for Environmental Information Disclosure (Trial)	State Environmental Protection Administration	8 February 2007	To promote and specify the obligations of environmental protection agencies and enterprises to disclose environmental information and protect the rights and interests of citizens, legal persons, and other organizations to obtain environmental information
Management Measures for Information Disclosure of Medical and Health Service Organizations (Trial)	Ministry of Health	3 June 2010	To disclose information on medical and health service organizations in conformance with the laws and to improve the transparency of the medical and health service work
Notice of the Ministry of Environmental Protection on Strengthening the Information Disclosure on Supervision of Environmental Pollution Sources	Ministry of Ecology and Environment	12 July 2013	To protect the rights and interests of citizens, legal persons, and other organizations to obtain information of environmental pollution sources using legal means and to issue guidance on public participation in environmental protection
Self-monitoring and Information Disclosure Measures for Key Enterprises under State Monitoring (Trial)	Ministry of Environmental Protection	30 July 2013	To specify self-monitoring and information disclosure for enterprises, urge enterprises to intentionally fulfill their legal obligations and social responsibilities, and to promote public participation
The Environmental Protection Law	Standing Committee of the National People’s Congress	24 April 2014	Enterprises and organizations shall disclose environmental information regarding environmental monitoring and public emergencies in accordance with the law; the public is entitled to the right to supervise environmental protection issues
Measures for Environmental Information Disclosure by Enterprises and Organizations	Ministry of Environmental Protection 19 December 2014	19 December 2014	Enterprises and organizations shall accurately disclose environmental information in accordance with the law; public participation, supervision, and management to be promoted
Measures for Public Participation in Environmental Protection	Ministry of Environmental Protection	13 July 2015	To protect the rights of citizens, legal persons, and other organizations in obtaining environmental information, as well as participating in and supervising environmental protection issues; develop avenues for public participation and promote the legal and orderly development of public participation in environmental protection

## Data Availability

The data presented in this study mainly derived from major media sources, office information and published studies shown in reference lists, which are openly available. Additionally, the data supporting public participation regarding the case study was obtained via questionnaire survey conducted by authors team, all relative results have been shown in Section 3.5.3.
